# Science teachers’ interactions with resources for formative assessment purposes

**DOI:** 10.1007/s11092-022-09401-2

**Published:** 2022-11-02

**Authors:** Ragnhild Lyngved Staberg, Maria Immaculata Maya Febri, Øistein Gjøvik, Svein Arne Sikko, Birgit Pepin

**Affiliations:** 1 Department of Teacher Education, Faculty of Social and Educational Sciences, Norwegian University of Science and Technology (NTNU), NO-7491 Trondheim, Norway; 2 Eindhoven School of Education, Eindhoven University of Technology (TU/e), P.O. Box 513, 5600 Eindhoven, Netherlands

**Keywords:** Formative assessment, Assessment for learning, Science education, Primary school, Curriculum resources, Documentational approach to didactics, Formative assessment practices, Teacher interaction with resources, Analogue and digital curriculum resources, Formative assessment resources, Inquiry-based classroom practices, In-service teacher education in science and mathematics

## Abstract

Using a case-study approach, we aim to understand how teachers interact with both analogue and digital resources in the science classroom for formative assessment (FA) purposes and their justifications for such interactions. The study was conducted in the context of a European Union project on FA in science and mathematics education. The case involved two Norwegian primary school teachers teaching their grades 5 and 7 students a series of science lessons on the topic “how to prevent microorganisms from spreading.” The data set consisted of lesson plans, classroom observations, pre- and post-interviews conducted with teachers, student tasks, post-interviews with students, and student work. We identified eight analogue and digital resources, which were used to employ five FA strategies. The strategies that were most commonly used related to “engineering effective classroom discussions” that elicited evidence of student understanding and “activating students” as autonomous learners and peer instructors. The teachers’ rationales for using the selected resources were mainly connected to their effectiveness, practicality, and relevance. Teacher interactions with the selected resources are described, and educational implications are discussed.

## Introduction

According to the research literature (e.g., Cairns, 2019), science educators agree that inquiry-based instructional approaches are suitable for science teaching. Over the last two decades, we have seen an increased focus on inquiry-based approaches at both research and policy levels (Hazelkorn et al., 2015; Rocard et al., 2007). Internationally, there has been increased attention to inquiry approaches in the science curriculum, with several European research and development projects aimed at raising the use of inquiry pedagogies in school (e.g., PROFILES, PRIMAS, Mascil). However, classroom studies have shown that there is still little emphasis on students’ development of exploratory skills and scientific ways of thinking (e.g., Heinz et al., 2017; Hume & Coll, 2010; Maaß & Artigue, 2013; Ødegaard et al., 2020). Many lessons would have the potential to become more exploratory, if students were allowed to participate more actively in asking questions, setting up hypotheses, and planning procedures (Ødegaard et al., 2020). However, as pointed out by Hmelo-Silver et al. (2007), these processes need to be appropriately scaffolded to ensure beneficial student learning outcomes, and it has been claimed (e.g., by Knain et al., 2019) that guidance through formative assessment (FA) is crucial for students’ success. It appears that teachers are more confident in teaching science through teacher-centered rather than student-centered inquiry (e.g., Kaya et al., 2021). To increase teachers’ confidence in enacting inquiry-based instruction, they need to be provided with inquiry experiences, and long-term professional development has been recommended (e.g., Chichekian & Shore, 2016). Moreover, several researchers have suggested that students’ development of inquiry competences in science education should be supported using formative assessment methods (e.g., Black et al., 2004; Grob et al., 2017; Hume & Coll, 2010).

Formative assessment (FA) has been given considerable attention in the international research literature (e.g., Bennett, 2011; Black & Wiliam, 2009). Because of its potential effectiveness, FA has been given high priority in educational policy in several countries. It appears that limited attention has been paid to embedding FA in subject teaching, including science education. Therefore, pre- and in-service teacher educators need to provide science teachers with FA teaching (and learning) experiences and to upgrade their skills, so that FA in science education can be successfully enacted (Espiritu et al., 2018). As a first step toward improving science-specific FA, there is a need to investigate how teachers apply FA resources and strategies in science lessons.

This study was conducted as part of the European project *FaSMEd*^1^ (2013–2016), which brought together eight countries to investigate the use of FA and inquiry approaches in mathematics and science education. For this paper, we focus on a set of science lessons on the topic of “how to prevent the spread of microorganisms.” We address the issue of how teachers implement FA in primary science classrooms, providing insights into the use of a variety of analogue and digital resources for FA purposes and how these resources can be used to enhance student learning. Our research question is as follows: *How do teachers interact with various resources in the primary science classroom for formative assessment purposes?*

In the subsequent section, we first explain the theoretical lenses used. Second, we present the methods before we go into the results section. Finally, we discuss our results and conclusions and offer recommendations.

## Theoretical lenses

In this section, we outline the three lenses we used to analyze our data: (a) FA, (b) curriculum resources and their quality, and (c) the documentation approach to didactics (DAD).

### Formative assessment (FA)

As reported in a number of meta-analyses (Black & Wiliam, 1998a; Hattie, 2009; Kingston & Nash, 2011), an effective method of enhancing student learning is the use of FA. In inquiry-based education approaches, it seems to be vital to facilitate situations in which students can obtain useful FA (Knain et al., 2019), as effective informal FA practices have been shown to be associated with student learning in scientific inquiry classrooms (Ruiz-Primo & Furtak, 2007). While efforts are being made to develop support for teachers who want to use assessment formatively (e.g., research into learning progressions, advances in technology, professional development, and coaching on FA), much work remains to be done in this regard (Trumbull & Lash, 2013). As educators and researchers have been investigating how teachers use assessments to inform instruction, it has become clear that “conducting FA is a complex process that requires extensive knowledge, including knowledge about student learning, domains of study, assessment, and pedagogy” (Trumbull & Lash, 2013, p. 14). However, there are indications that the implementation of FA in schools has, at best, been at general pedagogical level and that FA has been considered a general pedagogical resource (Sandvik & Buland, 2014, p. 10).

Classroom assessment is used for various purposes: assessment *for* learning, assessment *as* learning, and assessment *of* learning (Manitoba Education, Citizenship & Youth, 2006). Assessment *for* learning occurs throughout the learning process and provides the basis for determining what the teacher needs to do next to move student learning forward. Assessment *as* learning is the use of ongoing self-assessment by students to monitor their own learning. This is characterized by “students reflecting on their own learning and making adjustments so that they achieve deeper understanding” (Manitoba Education, Citizenship & Youth, 2006, p. 41). Assessment *of* learning refers to summative strategies designed to confirm what students have learned.

FA, as defined and described by Black and Wiliam (2009), is an assessment *for* learning: “all those activities undertaken by teachers and/or by their students [that] provide information to be used as feedback to modify the teaching and learning activities in which they are engaged” (p. 7). Assessing student knowledge *during* the learning process instead of (or in addition to) *at the end of* the learning process has proven to be helpful for learners and teachers (e.g., Hattie, 2014). Based on a research project examining FA processes in science classrooms, Bell and Cowie (1999) defined FA as “the process used by teachers and students to recognize and respond to student learning to enhance that learning, during the learning” (p. 101). In this study, we lean on the FA definitions of Black and Wiliam (2009) and Bell and Cowie (1999).

Researchers have claimed that FA can raise student achievement (e.g., Black & Wiliam, 1998a; Grob et al., 2017; Hattie, 2009; Hattie & Timperley, 2007; Ruiz-Primo & Furtak, 2007) and improve understanding, especially for low-achieving students (e.g., Black & Wiliam, 1998b, 2010). In a review of the literature on FA, Black and Wiliam (2009) identified five teaching strategies that are prevalent in effective FA (Table 1).

**Table 1:** Strategies of formative assessment (Black & Wiliam, 2009, p. 8)

No	Formative assessment strategies from teachers’ perspectives
1	Clarifying learning intentions and criteria for success
2	Engineering effective classroom discussions and other learning tasks that elicit evidence of student understanding
3	Providing feedback that moves learners forward
4	Activating students as instructional resources for one another
5	Activating students as the owners of their own learning

Black and Wiliam (2009) discussed FA, considering contributions from teachers, peers, and learners, as well as the learners’ processes. Our approach will focus on the teacher, and their use of analogue and digital FA curriculum resources during teaching.

### Curriculum resources and their quality

To clarify the notions of curriculum resources, we use (Pepin & Gueudet, 2014; Pepin et al., 2017; Pepin & Gueudet, 2018). In this body of work, curriculum resources are defined as all the material resources that are developed and used by teachers and students in their interactions with the subjects in/for teaching and learning, both inside and outside the classroom. Hence, curriculum resources would compose of the following: (1) text resources (e.g., textbooks, teacher curricular guidelines, websites, worksheets, syllabi, and tests), (2) other material resources (e.g., manipulatives and calculators), and (3) digital-based curriculum resources (e.g., interactive e-textbooks).

For most teachers, there is now a profusion of digital curriculum resources available on the web. These are considered important, particularly for teacher lesson preparation and classroom instruction, as well as for pupil learning (Pepin et al., 2017). There is potential for these materials to provide stimulating and meaningful learning experiences for students, as well as assessment opportunities for teachers to formatively provide feedback to their students. It has become clear that digital resources have the potential to embed assessment to provide feedback to students and performance data to a range of stakeholders, including students, parents, teachers, and administrators (Choppin et al., 2014). There are subtle and sophisticated forms of assessment in a number of digital applications (Choppin et al., 2014).

There are different ways of assessing the quality of curriculum resources (e.g., Forbes & Davis, 2010; Trgalova & Rousson, 2017; Ye et al., 2015; Gueudet et al., 2021). Moreover, the quality of curriculum resources also depends on the audience who uses the resources. For example, the usefulness of a resource depends on who uses it and why. In our case, the teachers would be the users, and they would use the curriculum resource to design lessons related to FA.

In the curriculum literature, we find criteria for evaluating the quality of the curriculum (e.g., Nieveen, 1999; Van den Akker, 2013, 2020). We use these quality criteria and adapt them to curriculum resources (Gueudet et al., 2021). In other words, leaning on van den Akker (2020), we conceptualize the quality of curriculum resources in terms of seven criteria: relevance, justification, consistency, practicality, effectiveness, scalability, and sustainability (see Table 2; Gueudet et al., 2021).

**Table 2:** Quality criteria for curriculum resources (adapted from van den Akker, 2020; Gueudet et al., 2021)

Criterion	Definitions
Relevance	The curriculum resource is relevant, and there is a clear and shared interest in it
Justification	The curriculum resource is supported by a credible knowledge base. Its design is based on state-of-the art scientific knowledge, or the resource is scientifically underpinned by the research literature in science education and in formative assessment
Consistency	The curriculum resource fits within the science curriculum, and it is coherent with its aims
Practicality	The curriculum resource is feasible in practice, for lesson preparation, or in class
Effectiveness	The curriculum resource provides the intended outcome for the teacher or students
Scalability	The curriculum resource can be used across various contexts
Sustainability	The curriculum resource is sustainable for schools/teachers/students over time

### Documentation approach to didactics (DAD)

Using the notion of resource for all the resources that are developed and implemented by teachers (and pupils) in their interactions with the subject in/for teaching, inside and outside the classroom, (Trouche et al., 2020) developed the concept of DAD, which explains and describes the processes involved when teachers (or students) interact with (curriculum) resources.

Taking a step back, Vérillon and Rabardel (1995) distinguished between an *artefact* (or resource) and an *instrument*, viewing the latter as a psychological construct and stating that no instrument exists in itself: “An instrument results from the establishment, by the subject, of an instrument relation with an artefact” (p. 85). An instrument is thus not given but has to be constituted by someone using it. The construction of an instrument through its use by a subject in a community of practice is called an *instrumental genesis* (Trouche et al., 2020; Trouche, 2004). Instrumental genesis contains two dialectical processes: *instrumentation* and *instrumentalization* (see below).

The DAD leans on the instrumental approach, whereby the artifact is the resource that becomes a document (instrument) during teachers’ interactions with the resource. In terms of processes, during the interaction with a particular resource or set of resources, teachers (and students) develop their particular *schemes of usage* with these resources. The outcome is the *document*; hence: *Resource(s)* + *scheme of usage* = *document.*

**Fig. 1:**
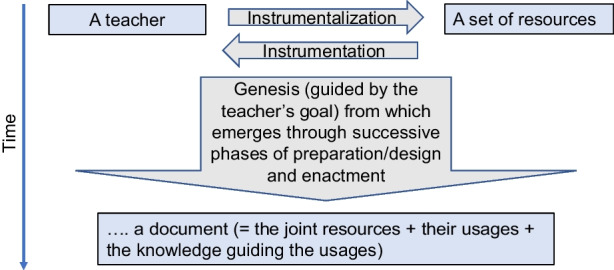
A representation of the notions and processes involved in DAD (reproduced from Trouche et al., 2018)

The DAD is particularly pertinent to viewing the use of resources as an interactive and potentially transformative process. This process works both ways: the affordances of the resource(s) influence the teachers’ practice (the *instrumentation* process); at the same time, the teachers’ dispositions and knowledge guide the choices and transformation processes between different resources (the *instrumentalization* process) (Fig. 1). Hence, the DAD emphasizes the dialectic nature of the teacher–resource interactions combining instrumentation and instrumentalization (Vérillon & Rabardel, 1995). These processes include the design, redesign, appropriation, or “design-in-use” practices (whereby teachers change a document in the moment and according to their instructional needs).

The DAD proposes a model of interactions between teachers and resources that has implications for teachers’ professional learning. While an enormous number of potentially suitable resources are provided online, the web does not provide suitable support for using these resources, especially their use in particular classrooms (e.g., the science classroom). Whether searching for tasks to supplement a given learning sequence or planning to formatively assess students’ work, teachers typically require professional support.

## Methods

Adopting a case-study approach (Yin, 2018), we conducted an in-depth examination of two teachers’ FA practices as part of a Lesson Study approach (e.g.; Elliott, 2019; Yang & Ricks, 2013). Hence, the case was one teacher, and the unit of analysis was one teacher and the teacher’s class. Due to the Lesson Study approach, the two cases were interdependent. The two teachers represent two perspectives, but the study mirrors collaborative practices in schools, in which one teacher’s practice cannot be studied isolated. The context was a series of science lessons on “how to prevent microorganisms from spreading.” In the following paragraphs, we outline the sampling of the case teachers and lessons included in the study, and we explain our data collection strategies and methods of analysis.

### Case teachers B and S

The selection of the two case teachers (Teacher B and Teacher S) (Table 3) can be categorized as convenience sampling (Cohen et al., 2018). Teachers B and S were selected from a group of six teachers involved in the *FaSMEd* project; they both volunteered to participate in the study and were chosen as illustrative cases. Both teachers worked in primary schools in a medium-sized city in Norway. At the time of the study, Teacher B taught a Grade 5 class with 19 students (10–11 years old), and Teacher S taught a Grade 7 class with 19 students (12–13 years old).

**Table 3:** Case teachers in this study

Teacher	Gender	Age (years)	Education^a^	Experience (years)^b^	School^c^
Teacher B	Female	30	Bachelor of education, 4 years, from the UK, specialization in English and literature	8.5	B (Grades 1–10, 264 students, 24 teachers, private, international, English as teaching language)
Teacher S	Female	44	General teacher education, 4 years, from Norway, master’s degree in special education	16.5	S (Grades 1–7, 592 students, 48 teachers, public)

^a^Formal education

^b^Number of years practicing as teacher

^c^Data retrieved from skoleporten.udir.no

Both case teachers were part of a 4-month professional development (PD) program within the *FaSMEd* project. Principles of FA, inquiry-based learning, and different technological resources and computer programs for FA were introduced to the project teachers during PD meetings at Norwegian University of Science and Technology. The resources for FA included the student response systems (SRSs) Socrative (socrative.com) and Kahoot (kahoot.com). The PD consisted of five half-day sessions at the university, with teaching sessions in school in between, and two lesson study cycles (one in science and one in mathematics) at two of the participating schools. Data taken from the lesson study cycle in science were used for the study reported in this paper. The planning of the experimental lessons for this study started at one of the PD meetings.

### Science lessons

The lesson study cycle in science consisted of a series of lessons—two at School B and three at School S—on “how to prevent microorganisms from spreading.” These lessons were selected because of their relevance to students (and their families) in case an epidemic occurs. The lessons were planned and conducted in Teacher B’s class and were subsequently redesigned for Teacher S’s class. In this way, Teacher S’ lessons built on the experiences of the lessons in Teacher B’s class, thereby constituting a second loop of the lesson study cycle. The lessons were planned based on the principles of FA and inquiry-based learning that had been introduced at the *FaSMEd* meetings at the university. At each school, the regular teacher (Teacher B or S) enacted the planned teaching, while the lessons were observed by the five other members of the *FaSMEd* teacher group and by the researchers from the university. Following completion of the lessons, the whole group of teachers and researchers met to reflect on the enacted lessons and to discuss possible redesigns. In particular, the lessons in Teacher B’s class were the foundation for the lessons in Teacher S’ class.

The lessons in question were about the spread of microorganisms through the air. The students were expected to explore how far the droplets that result from sneezing can spread and how best to prevent it from spreading by performing practical experiments and recording the results. The students had to formulate hypotheses about the most effective methods of preventing spreading; thereafter, they performed experiments in groups to test the hypotheses. The experimental part of the lesson consisted of students performing “sneezing” experiments using paper towels, paper rolls, rulers, tape measures, and spray bottles filled with water colored with food coloring. The students were supposed to measure how far the drops of colored water traveled when it was sprayed without preventing the spreading, as well as when different methods of preventing it were employed—for example, inhibiting the spread of the spray with an elbow, tissues, or a hand.

At School B, the lesson series ran over 2 days, totaling approximately 4 h. The lessons started with the teacher informing the class that they had received a letter from a boy who had caught a cold and was anxious about passing it on to his grandmother. He wanted advice on how to prevent his cold from spreading. The results of the experiments were recorded by the students on the smartboard, and they drew bar graphs on large sheets of paper.

At School S, the lesson series ran over 3 days, totaling approximately 6 h. At this school, the theme was put into a real-life context by the school principal writing a letter to the class asking for help to reduce sickness among the teachers. The students performed the same type of experiments as those done at School B to determine the best way to prevent the spread of microorganisms. The results were recorded using Excel spreadsheets, and the students were supposed to choose appropriate graphical representations using Excel. 

The students at both schools were expected to conclude their work by writing a letter to the boy or the principal, providing the results of their investigations.

### Data collection

Various kinds of data were collected to enable their triangulation: lesson plans (LPs) (two for Teacher B and three for Teacher S), classroom observations (COs) (e.g., audio recordings, observation sheets from all observers, and classroom photos) (two audio recordings for Teacher B and three for Teacher S), computer files of students’ work and screenshots of students’ work (SW) (24 from School B and 22 from School S), student post-interviews (SIs) (one group interview per class), and teacher pre- and post-interviews (TIs).

The teachers were interviewed individually prior to and after the lessons. The interviews were semi-structured (Cohen et al., 2018). The teachers were asked about their beliefs concerning FA, their use of technology in relation to FA, their experience with *FaSMEd* PD, and its impact on their teaching practice. To corroborate each teacher’s answers, semi-structured focus group interviews were conducted with one group of three to four verbally strong students per class after the lessons on microorganisms. The students were selected by their teachers. The students were asked about their experiences with the *FaSMEd* lessons and whether these lessons differed from the types of lessons they normally had. They were also asked to reflect on useful and difficult aspects of the lessons; whether these lessons helped their learning of science and, if so, in which ways; and whether they would prefer this type of lesson in the future.

### Data analysis

The collected data set was subject to a systematic thematic analysis (Braun & Clarke, 2006), which was performed in five steps (Castleberry & Nolen, 2018): compiling, disassembling, reassembling, interpreting, and concluding.

As the first step, audio recordings from the teacher and student interviews were transcribed. We examined these transcripts, LPs, and COs and identified all the resources that Teachers B and S used for FA purposes. We also identified not only how these resources were used but also the teachers’ arguments for and against their use. Students’ interviews and students’ work were used to validate the teachers’ use of resources.

After compiling and organizing the data set, it was separated for the purpose of creating meaningful groupings. Thus, in the second step, the identified resources were coded according to the five FA teaching strategies presented by Black and Wiliam (2009) (Table 1). Next, the teachers’ arguments for using the resources were coded inductively using empirically based codes identified upon reviewing the data. For example, Teacher B highlighted the value of the incorporated report in Socrative: “You can go into the report and see and then come back to that in a follow-up lesson because it gives you more of a chance to look over and see who is understanding.” This quote was coded as “Report included.” Both teachers highlighted that several resources were easy to use. For example, Teacher S stated, “The first time using Kahoot then they are *in* right away; they understand the concept immediately.” This resulted in the code “Easy to use.” All arguments were also linked to whether the referred resource was analogue or digital. All the emerging codes are presented in the “Results” section.

As the third step, we further analyzed the teachers’ arguments by identifying patterns in the initial codes. These codes were put into contexts with each other to create themes. For this third step, we used a deductive approach: Initial codes were sorted using the amended quality framework (Table 2) as a coding tool.

As a fourth step, we interpreted the thematic patterns across the data to place the themes in the larger context. Finally, we drew conclusions based on the previous analytical steps.

To ensure validity and inter-rater reliability, all authors first worked thoroughly through the data set individually. Subsequently, we met and went through the data together. Where the analyses did not coincide, we negotiated to arrive at a common interpretation. In the reflection sessions of the lesson study, COs were compared and discussed with all teachers and researchers involved. Use of and interaction with the resources was discussed and thus represents not only the researchers view, but also a common understanding within the group. We therefore regard these sessions as reliability checks.

## Results

In this section, we present the FA resources that emerged from our data, as well as how the two teachers interacted with these resources and rationalized their interactions. In the following section, we denote as FA resources all the material resources used by teachers for FA purposes. All electronic resources are referred to as digital, and all others are referred to as analogue.

### Identification of Teacher B’s and Teacher S’s FA resources

The analogue and digital FA resources used in the experimental lessons are provided in Table 4. As is evident in this table, the analogue and digital resources were nearly equally represented; however, because of the relatively high number of digital resources, we consider both classrooms to be technology rich. Most of the resources provided in Table 4 are general, while few are task-specific resources for the microorganism lessons. All the resources provided in Table 4—including the teachers’ FA strategies for each resource, as well as their rationale for using them—are explained in detail below.

**Table 4:** Analogue and digital resources used by the two teachers for formative assessment

	Analogue resources	Digital resources
Teacher B	Mini whiteboards, Post-it notes, “traffic lights ^a^,” students’ products (graphs on paper sheets and letter to boy), task-specific resources (task description, tape measure, ruler, and spray bottles)	Computer, iPad, interactive whiteboard (smartboard), Smart Notebook ^b^, SRSs (Socrative and Kahoot)
Teacher S	Mini whiteboards, Post-it notes, blackboard, task-specific resources (task description, tape measure, ruler, and spray bottles)	Computer, iPad, interactive whiteboard (smartboard), Smart Notebook ^b^, SRSs (Socrative and Kahoot), students’ products (graphs in Excel and letter to principal), task-specific resources (Excel and email), learning management system (It’s Learning)

^a^Color pencils in red, orange, and green

^b^Software for smartboards

### Identification of Teacher B’s and Teacher S’s FA strategies using the resources

**Fig. 2:**
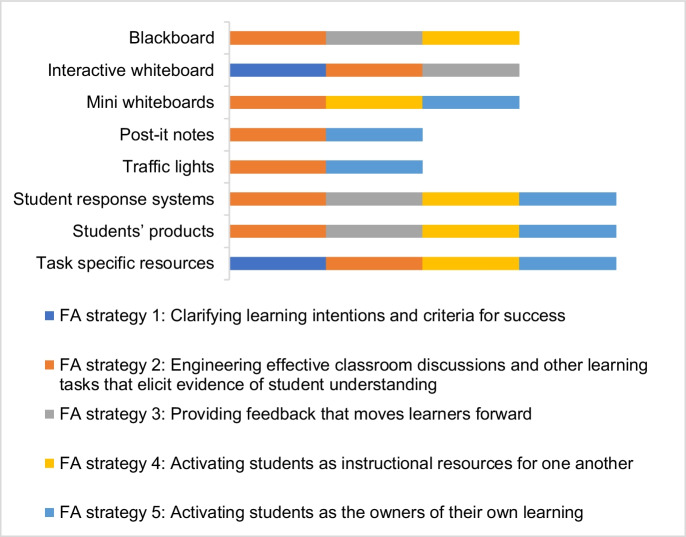
Identified FA strategies for the teachers’ FA resources. Strategy numbers are based on Black and Wiliam (2009) (Table 1)

For each of the resources found (Table 4), we identified which FA strategies (1–5, Table 1) the teachers applied using this specific resource. In Fig. 2, we provide an overview of all of these connections. When combining resources with strategies, we see that FA strategy 2 (engineering effective classroom discussions and other learning tasks that elicit evidence of student understanding) was most commonly used. Following strategy 2, strategies 4 (activating students as instructional resources for one another) and 5 (activating students as the owners of their own learning) were the most used strategies. These three strategies were used with 8, 5, and 6 different resources, respectively (Fig. 2).

As an illustrative example, the teachers used mini whiteboards to establish students’ pre-knowledge at the beginning of lessons (strategy 2) and during lessons to gain feedback on the students’ learning process (strategy 5). They also used this resource for peer-to-peer feedback (strategy 4) by having the students discuss each other’s writings and drawings on their mini whiteboards. Another analogue example is the Post-it notes, which students had to use as votes for the correct hypotheses (strategy 2). In addition, such notes were used as a general resource to provide feedback on what students thought and knew, with the planning of the next lesson in mind (strategy 5). An example of a digital resource is the smartboard, which both teachers used to present tasks and initiate classroom discussions (strategy 2). A task-specific digital resource was the student graphs in Excel, which were used for peer-to-peer feedback (strategy 4), the evaluation of one’s own learning (strategy 5), and the initiation of group discussions (strategy 2).

### Teacher B’s and Teacher S’s rationales for using the FA resources

From the pre- and post-interviews and the LPs, we gained insights into the teachers’ rationales for using the identified FA resources, which were validated through COs. Through open coding of the empirical data, we labeled the teachers’ arguments using 15 initial codes (Tables 5 and 6). By applying our quality criteria for resources (Table 2), we found that our initial codes could be sorted into seven themes corresponding to the seven quality criteria in Table 2. Some initial codes represented two or more themes because of the arguments given. For example, the initial code “Quick feedback” was given the theme “Practicality” because Teacher B highlighted the practical usefulness of the resource in a busy everyday life: “When we’re busy at school, the ideal is that you’ve got something really quick.” In addition, “Quick feedback” was placed under the theme “Effectiveness” because Teacher B stated that the resource was effective with respect to facilitating her students’ progress—“I’ve used it in different subjects, especially in math, for some kinds of little check-ups, like quick little things”—and that she “gets something valuable out of it.” As shown in Tables 5 and 6, most codes were associated with the three themes of “practicality,” “effectiveness,” and “relevance.” Teacher B (Table 5) used most arguments associated with “practicality” and “effectiveness,” while Teacher S (Table 6) focused on “relevance,” “practicality,” and “effectiveness.”

**Table 5:** Codes that emerged during the thematic analysis of Teacher B’s arguments. Initial codes appeared as a result of inductive coding, while themes were based on Table 2. Arguments were also sorted with respect to their connections to analogue (A) or digital (D) resources

Initial codes (15) (Teacher arguments)	Example quotes from Teacher B (B)	# Arg. from (B)*	Connected to analogue (A) or digital (D) resource	Related themes (7)
Teacher B				
** School context**—The resource is supported by the school context (courses and attitudes of leaders and teachers)	*Socrative and the other types of clicker system because they have been really well liked at school (B)*	1	D	**Relevance**
** Normal learning environment**—The resource is part of the normal learning environment	*When they have to talk to each other about their ideas and justify and to give their reasons, that’s a regular thing anyway (B)*	1	D	**Relevance**
** Online content**—The resource is convenient because content is available online	*Kahoot and Socrative…there’s already a lot of content available for those online (B)*	1	D	**Justification** **Practicality**
** Topic specific application**—The resource is topic specific	*I can see how it could fit certain topics better than others (B)*	3	D	**Consistency**
** Ease of use**—The resource is easy to use	*Socrative and the other types of clicker system because they … are really easy to implement actually (B)*	1	A and D	**Practicality**
** Technical challenge**—The resource implies a technical challenge	*It’s been a bit of a problem [in terms of] how to update everything (B)*	1	D	**Practicality**
** Time challenge**—The resource implies a time challenge	*Set up your own Socrative or Kahoot and things up; that does take time (B)*	3	D	**Practicality**
** Quick feedback**—The resource gives quick feedback	*I do see the value of using the technology now, especially when you can do a quick thing… (B)*	5	A and D	**Practicality** **Effectiveness**
** Report inclusion**—The resource generates a useful report	*…go into the report and see, and then come back to that in a follow-up lesson because it gives you more of a chance to look over and see who is understanding… (B)*	2	D	**Practicality** **Effectiveness**
** Motivation**—The resource promotes student engagement or motivation	*It’s perfect for getting kids motivated and interested and wanting to work (B)*	6	D	**Effectiveness** **Relevance**
** Cross-curricular application**—The resource is applicable across subjects	*Kahoot and Socrative…they can transfer across a lot of subjects (B)*	3	D	**Scalability** **Consistency**
** Flexibility**—The resource is applicable in different ways	*Kahoot…I can use it in lots of different ways (B)*	1	D	**Scalability**
** Long-term usefulness**—The resource is useful on a long-term basis	*Kahoot and Socrative…I think they’re more useful on a long-term basis, actually (B)*	1	D	**Sustainability**

*Number of arguments for Teacher B within each initial code

**Table 6:** Codes that emerged during the thematic analysis of Teacher S’s arguments. Initial codes appeared as a result of inductive coding, while themes were based on Table 2. Arguments were also sorted with respect to their connections to analogue (A) or digital (D) resources

Initial codes (15) (Teacher arguments)	Example quotes from Teacher S (S)	# Arg. from (S)*	Connected to analogue (A) or digital (D) resource	Related themes (7)
Teacher S				
** School context**—The resource is supported by the school context (courses and attitudes of leaders and teachers)	*We had a smartboard course (S)*	2	D	**Relevance**
** Availability**—The resource is available at our school	*We’ve got a smartboard in every classroom (S)*	2	D	**Relevance**
** Normal learning environment**—The resource is part of the normal learning environment	*So, if I have a day without a smartboard, then it will be a bit strange (S)*	1	D	**Relevance**
** Online content**—The resource is convenient because content is available online	*There are lots of apps that can go directly in [for IPad, smartboard], especially in science then (S)*	1	D	**Justification** **Practicality**
** Topic specific application**—The resource is topic specific	*… especially in science then (S)*	1	D	**Consistency**
** Ease of use**—The resource is easy to use	*The first time using Kahoot then, they are* in *right away; they understand the concept immediately (S)*	5	A and D	**Practicality**
** Technical challenge**—The resource implies a technical challenge	*There is someone who shows resistance to the technical part (S)*	2	D	**Practicality**
** Quick feedback**—The resource gives quick feedback	*These popular things like Kahoot, where we can get quick polls and such things (S)*	1	A and D	**Practicality** **Effectiveness**
** Reflection**—The resource initializes student reflection	*…but Socrative is more reflective, I think (S)*	4	A and D	**Effectiveness** **Relevance**
** Motivation**—The resource promotes student engagement or motivation	*I do see how motivating it is. The students become eager (S)*	4	D	**Effectiveness** **Relevance**
** Cross-curricular application**—The resource is applicable across subjects	*We see that we can use a lot of this into everyday life in different subjects as well (S)*	1	D	**Scalability** **Consistency**
** Flexibility**—The resource is applicable in different ways	*There are lots of possibilities (S)*	1	D	**Scalability**

*Number of arguments for Teacher S within each initial code

As we see from Tables 5 and 6, the teachers’ arguments for using FA resources in general were mainly connected to effectiveness (in terms of quick feedback, student motivation and reflections, and an incorporated report tool), practicality (in terms of being easy to use, available online content, technical challenges, and time challenges), and relevance (in terms of supportive school context, availability, links to the normal learning environment, reflection, and motivation).

Analogue resources were considered useful because of their practicality, effectiveness, and consistency. No negative arguments for analogue resources were mentioned. Digital resources were considered challenging because of their practicality (time challenge and technical challenge), but nearly all arguments regarding digital resources were considered positive. These arguments were connected to relevance (availability, supportive school context, and links to the normal learning environment), practicality (ease of use), effectiveness (quick feedback, reflection, and motivation), and scalability (applicability across subjects and in different ways).

### Teacher B’s and Teacher S’s interactions with the FA resources

In this section, we describe in more detail how the two teachers interacted with each of the identified resources in terms of FA. For each resource, we present how it was used, teachers’ arguments for using it, our observed rationale for its use, links to themes (Tables 2 and 5), and connections to FA strategies (Table 1, Fig. 2). Not all resources were used by both teachers; see Table 4 for a full overview of all resources used by each teacher.

#### Blackboard

##### Teacher S

Based on the COs of Teacher S, we know that the blackboard was used regularly to inform students and to facilitate discussion. This resource was part of the normal learning environment at School S and was used because of its availability, flexibility, and ease of use. As observed and referred to by Teacher S, notes or mind maps on the blackboard were also used because they initialized student reflections: “They had their own goals…[You] should see how much the students got from what they presented. Then they had different [methods]…Some had mind maps on the blackboard and made the students participate” (TI, pre-interview, Teacher S, p. 6). Referring to the Table 2 categories, the blackboard was used because of its relevance, practicality, effectiveness, and scalability. According to the interview of Teacher S, for FA purposes, the blackboard was used to engineer tasks, facilitate reasoning and argumentation, map students’ understanding, and encourage peer-to-peer feedback (FA strategies 2, 3, and 4; see Table 1 and Fig. 2). As a resource, the blackboard was well known to Teacher S, and she had ways to shape its use and knowledge to exploit its possibilities for her purposes—that is, it constituted a document in the sense of DAD.

#### Interactive whiteboard

Interactive whiteboards provide many opportunities for teachers. During the observed lessons, the case teachers did not use the other tools incorporated into the Smart Notebook software. In this case, the instrumentalization process of DAD means that the teachers viewed the smartboards as having many of the same constraints and affordances as regular blackboards. But still, the teachers reported new utilization schemes involving lesson planning and implementation.

##### Teacher B

Teacher B highlighted planning as a main advantage of the Smartboard: “The smartboard is on all day, every day when I’ve got lessons in there. (…) So, I think that has been the absolute best technology that we could have gotten. (…) All my planning really is done through the notebook files; I just have all my LPs there ready to open up” (TI, pre-interview, Teacher B, p. 8).

##### Teacher S

According to the teacher interviews, this resource is used every day: “So, if I have a day without a smartboard, then it will be a bit strange” (TI, pre-interview, Teacher S, p. 9). The arguments for using smartboards are that they are supported by the school context; for example, they are available (“We have a smartboard in every classroom,” pre-interview, Teacher S, p. 7), and as mentioned above, they are part of their normal learning environments. Technologically, the interactive whiteboards support making lessons available to be opened up at any time when appropriate.

In terms of FA, interactive whiteboards (smartboards) were mainly used as traditional blackboards to present learning goals and record and present results (CO at Schools S and B). The teachers also mention technical challenges—for example, regarding updates. In addition to these teacher arguments, we state that teachers used smartboards because of their flexibility (the resource is applicable in different ways) and their applicability across subjects. In terms of the Table 2 categories, we see that our case teachers used this resource because of its relevance, practicality, scalability, and consistency. For FA purposes, smartboards were used to present and clarify learning goals, record the results of students’ experiments, present results, and explain results (FA strategies 1, 2, and 3; see Table 1 and Fig. 2).

#### Mini whiteboards

Mini whiteboards were used routinely by both teachers for FA purposes: The students brought one board each when they entered the classroom (CO).

##### Teacher B

Teacher B established students’ pre-knowledge on the concept of microorganisms and related diseases using mini whiteboards before she moved the lesson further toward investigation (CO):

You are going to write down your answers on the whiteboard. OK. Are you ready? What are the three types of microorganisms?...Three, two, one…Let me see. What did you get, Student x?…Next one: How can viruses spread? Let me have a little look around. Good job, you’ve [got] lots of good ideas around here.” (CO, School B)

By the time the students started the experiment, they had some ideas written on the whiteboards regarding how to prevent spreading.

##### Teacher S

The students at School S were asked to write on the mini whiteboard their suggestions on the most effective way to prevent the spread of microorganisms, as well as their arguments on the pros and cons of each method. The following is an example of the students’ answers: “The tissues are best because they (microorganisms) do not spread as fast. The inconvenience is that one does not have them (tissues) all the time. The advantages are that one can change to a new one (once it is used)” (CO, School S).

Teacher S appreciated the possibility of seeing answers from all the students in the class instead of having only one answer to each question from an eager student raising their hand:

To visualize this, we can set up a simple calculation. For example, 20 students multiplied by four questions = 80 answers. One student multiplied by four questions = four answers. If we repeat this with 3 hours of science a week, we get 240 responses instead of just 12. (LP, Teacher S, p. 2)

Teacher S also highlighted the mini whiteboard’s influence on students’ motivation: “The students’ direct involvement and motivation are getting higher, and it is easy to check the students’ understanding and knowledge. It is important that students experience recognition of their own opinions” (LP, Teacher S, p. 2). In addition, she stated that mini whiteboards were effective and that she wanted to continue their use: “I am using those whiteboards actively myself, and I see that it is effective, and I would keep that” (TI, post-interview, Teacher S, p. 1).

The teachers used mini whiteboards at the beginning of lessons to establish students’ pre-knowledge and during the lessons to gain feedback on the students’ learning process. They also utilized the resource for peer-to-peer feedback by engaging the students in discussions about each other’s writings and drawings on their mini whiteboards.

The teachers’ arguments for using mini whiteboards were mainly their potential for rapid feedback, as it gave them a quick glance at students’ answers, and it activated all the students. The small size of the mini whiteboards forces the teachers to rethink what kinds of questions to ask in the classroom so that it is possible for the students to give answers on the mini whiteboard and subsequently for the teacher to notice the students’ answers at a glance. This instrumentation–instrumentalization process, which is guided by the teachers’ goal, results in a document in the DAD sense.

Based on our data, we state that mini whiteboards were used because they were part of the normal learning environment in both classes owing to their availability, flexibility, cross-curricular potential, ease of use, and the fact that they allowed for reflection by all the students. Considering the Table 2 categories, we found that mini whiteboards were used as resources because of their relevance, practicality, effectiveness, scalability, and consistency. For FA purposes, this resource was used to establish students’ pre-knowledge, clarify learning goals, determine what students know and can do, and encourage peer-to-peer feedback (FA strategies 2,4,5, see Table 1, Fig. 2).

#### Post-it notes

Post-it notes were handed out by both teachers to individual students or student groups to collect their ideas and answers.

##### Teacher B

Teacher B let the students vote for the correct hypotheses using Post-it notes (CO, School B). Teacher B also facilitated students’ use of Post-it notes to construct a bar chart.

##### Teacher S

Teacher S let the students put the notes with their answers by the door (TI, pre-interview, School S, p. 6). These Post-it notes served as exit notes when the students left the session (CO, School S); this enabled Teacher S to collect them before she left the classroom, read the students’ feedback, and plan her next session accordingly.

Post-it notes are inherently easy to use. For FA purposes, the teachers found different ways to use the Post-it notes, as described in this case. This means that we could directly observe the instrumentalization process (cf. DAD), for example in the bar chart situation. Based on COs, this resource was part of the normal learning environment at both schools, and it was used because of its availability and ease of use, as well as the fact that it allowed for student reflections and was a flexible resource that was also applicable across subjects. With reference to the Table 2 categories, we state that Post-it notes were used because of their relevance, practicality, effectiveness, scalability, and consistency. For FA purposes, Post-it notes were used to determine what the students thought and knew and to provide feedback (FA strategies 2 and 5; see Table 1 and Fig. 2).

#### Traffic lights

Traffic lights as a resource are color pencils in red, orange, and green, which the teacher asks individual students to use to assess their own understanding.

##### Teacher B

Teacher B talks about how she uses the traffic lights in class:

Underline it in red if you really don’t feel you’ve got this. Or orange if you think you understand it. Or green if you think, *Yeah, I can do that*...And then I can quickly look at the books and see: Oh yeah, these kids think they know this. At least they feel comfortable doing it or need some more work there. That is a quick way of being able to check up on them. (TI, post-interview, Teacher B, p. 11, and CO).

By activating her students to use these three colors, Teacher B asked them to label their understanding as good, medium, or bad. Through the lens of DAD, this may be considered the instrumentalization process in that the teacher redesigns or transforms the common traffic light sign into an FA document. Teacher B’s argument for using this resource was its usefulness in providing her with quick feedback. Based on COs and interviews, we state that this resource was part of the normal learning environment in this class and that it was used because of its availability, ease of use, and applicability across disciplines. In general, and in relation to the Table 2 categories, this resource was used because of its relevance, practicality, effectiveness, scalability, and consistency. For FA purposes, traffic lights were used as a form of self-assessment to elicit evidence of students’ understanding and to activate them as owners of their own learning (FA strategies 2 and 5; see Table 1 and Fig. 2).

#### Student response systems (SRSs)

LPs, interviews, and COs documented the two teachers’ use of SRSs, such as Kahoot and Socrative. Socrative was introduced as a new resource for both teachers through this project.

##### Teacher B

Teacher B saw SRSs as more advantageous than whiteboards for use in different subjects and in many ways—for instance, to “follow up” or “check up” on students’ work at a glance:

I do see the value of using the technology now, especially when you can do a quick thing, go into the report and see, and then come back to that in a follow-up lesson because it gives you more of a chance to look over and see who is understanding this and who isn’t in a way you don’t get when you’re doing a very quick form of assessment (…) on whiteboards. (TI, post-interview, Teacher B, p. 6).

SRSs have an element of competition and allow the students to be interactive. Because of this, Teacher B found the SRSs motivating for the students:

It is motivating, I think, and that is a big added value really for me—that you get kids that are often not inspired by reading or writing and doing things that way, to get inspired by the use of technology. (TI, post-interview, Teacher B, p. 6, referring to interactivity and competitiveness).

Socrative allows students to answer multiple-choice, closed, and open-ended questions. It also allows the answers to be saved by the end of the lesson and to be retrieved the next day to start the discussion, as observed for Teacher B (CO). The report generated by Socrative allowed Teacher B to adjust her course of action for the next lesson, as suggested by her reflection: “When we had that lesson on a Thursday, and then I had a chance to look at it on Thursday afternoon, and then I could guide my questioning to get a bit more out of it. That was very useful.” (TI, post-interview, Teacher B, p. 6).

Teacher B used Socrative to facilitate self-assessment, for instance, when the students were asked at the end of the experiments to answer open-ended questions to enable them to self-evaluate whether their own hypotheses were confirmed and why. For example, one student who had thought that the hand would offer the best protection wrote, “No, it [my hypothesis] was wrong because a hand is one of the worst,” while another student acknowledged the correctness of his or her prediction: “Yes, my prediction was correct. I made the prediction because the tissue prevents the sneeze and cough from reaching anyone.” The students were honest in their self-evaluation and had no problem admitting when their hypotheses were not confirmed by the experiments.

Teacher B also used Socrative as a base for peer-to-peer feedback (CO, TI, post-interview, Teacher B):

Being able to use the results from Socrative to base their discussions on has been really good because they have something concrete, and they have time to think about that and think about their own responses before they discuss [them] with their peers as well. (p. 10).

The students from School B confirmed that they had used Socrative for the first time during that lesson on microorganisms. They had had some prior experience with Kahoot. The students said that they found Kahoot or Socrative useful sometimes, stating, for instance, “The teacher can find out from your scores how much you already know about it” (SI, 04:20). In contrast, they also said that “sometimes you just guess, and then the software is not really useful” (SI, 04:30). Thus, they confirmed Teacher B’s enthusiasm for the new software Socrative with regard to effectiveness. As confirmed by the students, Teacher B often uses Socrative or other SRSs at the beginning of lessons to assess the students’ pre-knowledge (SI, School B), as well as at the end of lessons to assess how much the students have learned during the lessons (SI 05:00, LP, and CO, School B).

##### Teacher S

Teacher S was familiar with Kahoot, but was introduced to Socrative during the PD program. As teacher S put it: “Kahoot I have been using for a long time myself, but I do not know, really, what the learning outcome is. But Socrative is more reflective, I think. (TI, post-interview, Teacher S, p. 2). Teacher S appreciated the SRSs, but were aware of some challenges connected to how to use it to enhance student learning.

The teachers appreciated the system (and other types of SRSs) for several reasons. They felt that these systems were user-friendly, provided them with quick feedback, allowed student reflection, and increased students’ motivation. They also pointed to the flexibility of SRSs and to the fact that they were applicable across subjects, useful on a long-term basis, and convenient because of their available online content, which meant that they did not have to create all the questions from scratch. Although the teachers considered many advantages of using Socrative (and SRSs in general), they also mentioned some challenges—for example, the occasional lack of Internet stability, technical challenges (“Computers did not work”) (TI, pre-interview, Teacher B), time challenges (“log in”), and the fact that questions had to be prepared in advance (LP, CO, School B). Teacher B could also see how SRSs fit certain topics better than others.

Referring to Table 2, we found that the case teachers used SRSs as a resource owing to their justification, practicality, effectiveness, relevance, scalability, and sustainability. For FA purposes, SRSs were used for self-assessment, as an opinion poll, to capture pre-knowledge, to assess what students knew or had learned, and to encourage peer-to-peer feedback (FA strategies 2, 3, 4, and 5; see Table 1 and Fig. 2), as illustrated in the examples above. Through the lens of DAD, we see that the availability of SRSs forces teachers to carefully construct questions that enable the quantitative assessment of whole-class student understanding or qualitative responses that can be directly addressed in (future) lessons.

#### Students’ products

Teachers B and S regularly used students’ products as a basis for student reflections and classroom discussions.

##### Teacher B

Teacher B talked about how presentations could be used for FA purposes:

When they’ve done presentations, I usually give them a little active learning sheet, where they have to note down key points from the presentation and then give some feedback based on that. And we’ve talked about “What is good feedback?”—not just “It was great; I really loved the presentation.” Like, you’ve got to give them some kind of thing that they can improve and one thing that you thought was really good. (TI, post-interview, Teacher B, p. 11)

##### Teacher S

Teacher S asked her students to comment on each other’s presentations (CO, School S) and said, “Then I got such good conversations, and the students were high; they discussed, and I was actually superfluous. It was absolutely amazing” (TI, post-interview, Teacher S, p. 3). In this way, Teacher S utilized the students’ products to engage peers in discussions and comments, thereby turning the student product into a resource for FA. Referring to DAD, the document in this case is the student product, together with the utilization scheme of creating peer engagement and peer-to-peer feedback.

Teacher S’s students stated that they often have to present results and give each other feedback (peer feedback) and that they learn a great deal from these exercises. They stated that they learn from seeing what other students have done, by having to think about how this can help improve their own presentations, and by having to give feedback to others because it requires them to think carefully about the subject (SI, pp. 6–7). This confirms Teacher S’s use of student work as FA strategies 4 and 5 (Table 1), as well as the rationale connected to effectivity in terms of learning outcome.

Based on COs and interviews, we saw that all the students’ products from these lessons were used as FA resources because they allowed for reflection, were flexible (applicable in different ways), were cross-curricular (applicable across subjects), and promoted student motivation. Considering the Table 2 categories, we state that students’ products from the observed lessons were used by the case teachers because of their effectiveness, relevance, and scalability. For FA purposes, the students’ products were used as resources to capture knowledge, supervise the learning process, serve as a basis for continually running dialog to determine where students do well and where they need help, provide feedback, encourage peer-to-peer feedback, and promote student self-assessment (FA strategies 2, 3, 4, and 5; see Table 1 and Fig. 2).

#### Task-specific resources

In the observed lessons, task-specific resources were the task description (letter from boy or principal), tape measure, ruler, spray bottles, Excel software, and e-mail. Teacher B used analogue task-specific resources only and did not discuss these regarding FA purposes, so in this paragraph, we mainly focus on Teacher S and her use of digital task-specific resources.

##### Teacher S

Teacher S introduced students to Excel to facilitate students’ understanding and increase their motivation. Teacher S’s students confirmed that Excel was a new resource being used during these lessons. They stated that their main use of computers so far had been to design presentations (e.g., using PowerPoint), write texts, and find information on the Internet. For writing mathematics, they found it better to use pencil and paper and to make big posters. Thus far, they had not used computers nor technology in mathematics, but after this session, they could see the advantage of using spreadsheets to make diagrams, for example. They stated that having diagrams on the computer also facilitated their understanding:

You can make, like, line diagrams and such things. Can explain it, like we did in this lesson. That is good; it makes it easier to remember. You see it, and remember the name of the diagrams, and can explain what they are good for…which diagram is better for different situations. (SI, p. 4)

This confirmed Teacher S’s rationale regarding effectiveness in terms of motivation. Each of the task-specific resources demands its own instrumentalization process, resulting in its own documentational genesis. For example, the Excel spreadsheet is originally a general software that the teacher utilized for constructing tables and graphs showing the spread of microorganisms.

The task-specific resources were used to present the task, perform the experiment, and present the results. Based on COs, we state that task-specific resources were used because of their flexibility, usefulness on a long-term basis, and potential for increasing students’ motivation. Some of the resources were also associated with time challenges (what time to buy and collect) and technical challenges (For example, in Excel, such as how to use the various buttons).

In terms of the Table 2 categories, these task-specific resources were considered useful or challenging because of their sustainability, scalability, and practicality aspects. For FA purposes, these resources were used to clarify learning goals, map students’ skills, present the results of students’ work, supervise further progress, and monitor student dialogue to determine where students do well and where they need help (FA strategies 1, 2, 4, and 5; Table 1 and Fig. 2).

## Discussion

FA is a complex process that requires extensive knowledge, and previous research implies that it has been considered a general pedagogical resource (Sandvik & Buland, 2014). We know that students’ learning outcomes benefit from FA (e.g., Black & Wiliam, 1998a; Hattie, 2009; Kingston & Nash, 2011), and several researchers have suggested that students’ development of inquiry competences through FA methods should be supported (Hume & Coll, 2010; Ruiz-Primo & Furtak, 2007). Decristan et al. (2015) showed that students’ science understanding could be improved through embedded FA when combined with sufficient levels of global factors of classroom process quality (supportive climate and cognitive activation). To ensure effective instruction and, consequently, satisfactory learning outcomes, teachers need to combine specific teaching practices with high classroom process quality (Decristan et al., 2015). Therefore, there is a need to investigate science teachers’ classroom practices to acquire knowledge on science-specific FA strategies. The purpose of this study was to investigate teachers’ interactions with resources for FA purposes in science education.

Black and Wiliam (2009) presented several key strategies referring to the quality of implementing FA in a curriculum. Addressing these key strategies of FA when providing feedback has a major impact on student learning (e.g., Hattie, 2009; Hattie & Timperley, 2007). Kingston and Nash (2011) addressed the efficacy of FA, arguing that much of the variation in effect sizes might be related to the specific type of feedback provided within the formative condition. Based on their review, they have recommended that future research capture details on the types of feedback with clear descriptions of the forms and key features of the FA. Thus, in this study, we addressed teachers’ FA strategies and interactions with a set of FA resources.

On the topic of microorganisms, our case teachers utilized a large range of approaches for FA purposes. As other authors have reported (e.g., Grob et al., 2017), our case teachers appeared to favor bottom-up strategies, whereby they develop their assessment strategies themselves rather than receiving help externally. None of the selected resources were preloaded with specific content; they were tools for content creation. Most of the tools were made for learning (e.g., SRSs), but some had a general character that had to be adapted to learning situations (e.g., Post-it notes). Because of the general characteristics of the resources, the teachers were able to exploit the potential and adapt to science needs—for example, using Post-it notes in the process of selecting hypotheses. The students were expected to decide which hypothesis they thought was best and vote with their Post-it notes on the board, which gave the teacher an overview of all the students’ thoughts. During students’ preparation of graphs, task-specific resources, such as rulers and measure tapes, gave them an impression of the students’ mathematical and scientific skills.

The analogue mini whiteboards were commonly used in both schools, while the digital SRS Socrative was new for the teachers. These two resources were especially valuable for FA in science. The first was the mini whiteboard because it was useful for sketching, as well as for making representations and simplifications of processes in nature, which the teacher perceived as central to learning and understanding science. The second was Socrative because it offered the possibility of working with open-ended questions, which is a central part of inquiry-based approaches. In this study, these two resources were used in an interesting way: to establish students’ knowledge of and skills in science practices (Crawford, 2014) (in this case, the hypothetical-deductive method). The students came up with hypotheses and explanations for them. They evaluated their hypotheses and explanations in light of others’ explanations and their own experiments, all by using mini whiteboards, Socrative, and a peer.

Overall, all the FA resources considered in this paper were readily analyzed in light of the DAD. We identified the documents emerging from the teachers’ instrumentalization processes when they interacted with the FA resources. In doing so, we gained insights into how teachers utilized different FA resources and made them into their own ‘documents’ through the processes of instrumentalization and instrumentation. At the same time, the teachers were shaped by the resources they used and their affordances according to the quality of the resource(s) for the teachers’ needs. For the teachers, these processes were part of their PD and will be helpful in the future appropriation of FA resources.

The blackboard was used in a traditional way—that is, for writing notes and figures, including mind maps—but was also used to collect Post-it notes to create bar diagrams. The interactive whiteboard provides numerous possibilities for engaging learners and presenting content, including prefabricated resources. Our case teachers used some of the inherent affordances, such as preparing ready-made lessons. The fact that teachers need time to learn how to utilize more of the software included in interactive whiteboards may indicate that the instrumentation process is demanding.

We note that there is a difference between the use of analogue and digital resources. The analogue resources, such as traffic light or Post-it notes, were utilized by the teachers in new and creative ways, transforming them into FA resources that were not inherent in the resources itself. In contrast, the digital resources, such as SRSs and interactive whiteboards, were used more or less as intended by the manufacturers.

## Conclusions

From our data analysis, in practical terms, we argue that both analogue and digital resources were important (and used by our teachers) for FA teaching strategies. For the teachers, it was not the analogue or digital nature that mattered, but rather the resources’ perceived effectiveness with respect to FA strategies.

In theoretical terms, particular analogue and digital resources could be connected to

1. The teachers’ perceptions on the “quality” of the resource/s;
2. The teachers’ reasoning for the resources’ use; and
3. Particular teacher FA strategies.

Whilst (1) connects to the quality framework, (2) was related to teachers’ usage schemes of the DAD, and (3) to particular FA strategies. In other words, by looking through the lens of “resources” (Trouche et al., 2020), we could make links between three theoretical lenses: the quality of curriculum resources (van den Akker, 2020), the instrumentation and instrumentalization processes of the DAD (Trouche et al., 2020), and FA strategies (Black & Wiliam, 2009). We could use the three lenses in a productive way to understand teachers’ integration of digital resources for FA purposes.

In the process, we could, in effect, illustrate the instrumentation and instrumentalization processes: The perceived quality of the resources influenced the teacher’s decision-making about the use of the resource (instrumentation), at the same time as the teacher’s understanding and belief regarding FA strategies shaped the resource, to provide an instrument for the preferred teaching strategies. The DAD was helpful in identifying teachers’ interactions with the resource(s). Both the instrumentation and instrumentalization processes became evident in the same way as the teachers chose, used, and shaped the resources according to their beliefs (and experiences) regarding FA strategies (the utilization schemes were influenced by the teachers’ knowledge and beliefs of FA). The nature and affordances of the resources also shaped the teachers’ decision-making (e.g., whether and how to use the resources).

In summary, it is argued that this study provides insights into how analogue and digital FA resources can be utilized in science lessons. The in-depth character of the study, and the detailed analyses of the two cases, allowed for insights into science teachers’ perceptions and decision-making regarding the use of digital resources for FA strategies. According to Bennett (2011), FA is still a work in progress both conceptually and practically. There seem to be few “tested” FA resources specific to science, and hence, we need further studies on FA implementations in the science classroom, and the digital resources beneficial for such strategies. At the same time, we need investigations of students’ learning experiences in these learning environments.

## Limitations of the study

Having conducted a small-scale qualitative study, we acknowledge the limitations of the study. Due to the small number of cases, it is clear that one cannot generalize the findings. However, the fact that one of the case schools in the study was an international school could provide better potential for the results to be valid in other countries and different contexts. In addition, due to the COVID-19 pandemic, this particular topic area has become particularly relevant (which has implications for practice): students attempting to understand national and regional measures to contain and limit the spreading of the virus.

Further, the six teachers involved in the study were all part of the *FaSMEd* project. Pragmatism led us to follow only two of these teachers, and since teacher B and S volunteered, they would be our cases for recordings and interviews. The two sequences originated from the joint *FaSMEd* meetings, but even though they built on one another, various tools were chosen by the teachers, for FA purposes. The science lesson in Teacher S’ group of students was redesigned with experiences from Teacher B, but in spite of the interdependence of the planning, we considered the lessons as different cycles of the lesson study model, that is, treating them as two different cases.

## Data Availability

All data and materials are available upon request.

## Footnotes

1 *FaSMEd–Raising Achievement through Formative Assessment in Science and Mathematics Education* (https://microsites.ncl.ac.uk/fasmedtoolkit/)*.*

## Code availability

All codes used for analyzing the data are given in the results section. No software application was used in the coding process. All data and materials support our published claims and comply with field standards.
